# Variations between package inserts regarding gingival overgrowth causing drugs

**DOI:** 10.1007/s00210-025-04845-x

**Published:** 2025-11-27

**Authors:** Philipp Friedrich Georg Ried, Roland Seifert

**Affiliations:** Institute of Pharmacology, Hannover Medical School, Carl-Neuberg-Str. 1, D-30625 Hannover, Germany

**Keywords:** Drug-induced gingival overgrowth, DIGO, Calcium channel blockers, Immunosuppressants, Anticonvulsants, Adverse drug reaction, Package inserts

## Abstract

Drug-induced gingival overgrowth (DIGO) is an increasingly relevant topic among dentists, physicians and patients as well. Due to high prescription rates of calcium channel blockers, anticonvulsants and immunosuppressants, the prevalence of this adverse effect should not be underestimated. In this paper, we compare the three groups of active substances mentioned and work out the drugs with the highest risk of triggering DIGO. Additionally, we compared package inserts (PIs) of the drugs in question to identify differences and missing references to this adverse drug reaction (ADR). It is a well-known problem that package inserts sometimes contain different information about adverse drug reactions and their frequencies. To find out what prevalence can actually be expected, we analysed various package inserts for different drugs that are known to cause DIGO. Moreover, we compared package inserts with summary of product characteristics (SmPCs) as an example. It turned out that both pieces of information shed light on the circumstances and problems of DIGO with roughly the same degree of imprecision and incompleteness. Furthermore, the given information in SmPCs are sometimes even worse than in the PIs. To find out the prevalence with which the various drugs potentially trigger DIGO, recent prescription figures must be taken into account. Using current data from the Arzneiverordnungsreport (drug prescription report, AVR) 2023 and the prevalences stated in PIs, we performed a model calculation of the expected number of patients in Germany developing DIGO per year. It turned out that substantial differences in the frequencies and the reported adverse drug reactions in the different package inserts can be found. Apart from this, the compared SmPC do not contain any further information on DIGO and its frequency. Surprisingly, long-term therapy with valproic acid is associated with the highest rate of DIGO compared to the other drugs. Despite the lower number of prescriptions, significantly more cases of DIGO must be expected with valproic acid therapy than with amlodipine or phenytoin, for example. To summarise, there are significant differences in the content of package inserts from different manufacturers. As a source of information for healthcare professionals, the SmPC unfortunately does not provide any detailed information on DIGO. In patients receiving long-term therapy with valproic acid, particular attention must be paid to the development of DIGO to initiate therapy at an early stage.

## Introduction

Gingival overgrowth, previously described as “gingival hyperplasia” or “gingival hypertrophy”, is a common adverse reaction caused by many different drugs. Anticonvulsants, calcium channel blockers and immunosuppressants are the groups with the highest risk of developing this undesirable effect (Brown and Arany 2015). But not only drugs can cause this condition, among others being systemic conditions, hereditary factors, allergies, local irritant factors, hormonal changes and vitamin deficiencies (Beaumont et al. 2017; Moffitt and Cohen 2013).

The underlying mechanisms causing this condition are not yet fully understood. In general, the following cascade is used to explain the excessive overgrowth. Drug-induced blocking of sodium and calcium channels leads to an insufficient ion influx into the cell, which causes an intracellular deficiency of folic acid. This is followed by a disturbed activation of collagenases, which in turn leads to reduced dismantling of connective tissue and an imbalance between synthesis and degradation of extracellular matrix (Droździk and Droździk 2023).

Due to the extensive swelling of the gums, patients suffer from an unesthetic appearance, insufficient dental hygiene aggravating the swelling, pain, bleeding and masticatory dysfunction (Bharti and Bansal 2013). Increasing inflammation of the gums leading to periodontitis culminating in systemic inflammation and potentially tooth loss are complications (Chojnacka‐Purpurowicz et al., 2022).

Possible therapeutic approaches include improvement of dental hygiene, including scaling and root planing (SRP) as well as regular professional cleaning, modification or withdrawal of the causative medication if possible and reasonable and surgical intervention and removal of the excessive tissue to create hygienic conditions (Tungare and Paranjpe 2022). Unfortunately, drug-induced gingival overgrowth (DIGO) has a high recurrence rate, which results in regular surgical tissue removal every 12–18 months (Zoheir and Hughes 2019). Further treatment options include topical application of folic acid and systemic intake of antibacterial agents like azithromycin (Arya et al. 2011; Fuchs et al. 2019).

The best-known drugs to cause DIGO are phenytoin, nifedipine, ciclosporin and amlodipine (Brown and Arany 2015). Diltiazem, verapamil, valproic acid and carbamazepine are less familiar in this context. Reliable prevalence rates are hard to find because of the wide range that is reported from different sources describing one and the same substance (Droździk and Droździk 2023).

In principle, the frequency with which adverse drug reactions occur is summarised in six different categories. These categories are “very common”, “common”, “uncommon”, “rare”, “very rare” and “unknown”. The corresponding frequencies of affected patients are “ > 10%”, “1–10%”, “0.1–1%”, “0.01–0.1%”, “ < 0.01%” and “not assessable”. The categorisation corresponds to the classification of the Bundesinstitut für Arzneimittel und Medizinprodukte (BfArM) (n.d.) and is shown in Table 1 (Broich 2015).

**Table 1:** Illustration of the prevalences for adverse drug reactions as defined by the “BfArM” (Federal Institute for Drugs and Medical Devices)

BfArM definitions	Prevalence
Very common	> 10%
Common	1–10%
Uncommon	0.1–1%
Rare	0.01–0.1%
Very rare	< 0.01%
Unknown	Not assessable

In principle, all relevant information regarding the adverse drug reactions of a particular active substance, as well as the associated prevalence with which this ADR occurs, should be included in the package inserts of the medicinal products. Unfortunately, despite numerous studies that illustrate this circumstance, there are repeatedly discrepancies in the PIs of different manufacturers with identical active ingredients (Arning and Seifert 2024). This not only leads to confusion for patients, but also to a loss of trust in the information provided. Furthermore, package inserts are often not easy to understand, which can possibly lead to misunderstandings and prevent patients from reading them carefully (Arning and Seifert 2023).

Therefore, this study tries to depict, as comprehensively as possible, what drugs are most likely to cause DIGO and what prevalences can be expected. To tackle this task, we analysed various package inserts of drugs from different manufacturers containing the same active substance.

## Material and methods

### Analysis of adverse drug reactions

**Fig. 1:**
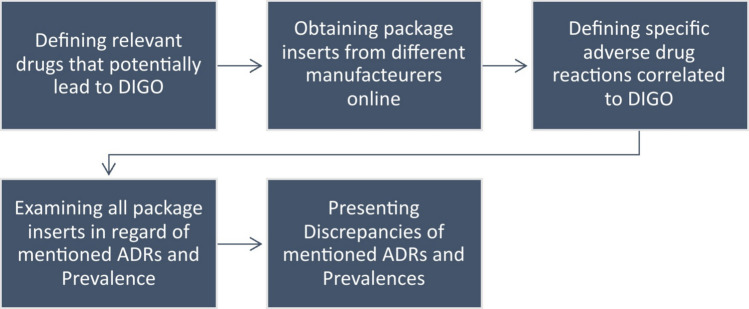
Flowchart describing the underlying methods used for this paper about drug-induced gingival overgrowth

Package inserts of suspected drugs were searched for gum specific adverse reactions like “gingival overgrowth”, “gingivitis”, “stomatitis” and “gum hypertrophy”. Specific reactions and expected prevalences were collected and documented in tabular form. The methodological approach is shown in Fig. 1.

Twenty-one pharmaceutical manufacturers were selected, and the package inserts provided for certain active ingredients were analysed. Among the pharmaceutical manufacturers, those whose PIs were available online at the time of the study were chosen. There were no specific exclusion criteria for the individual manufacturers.

By analysing PIs for different active ingredients from a single manufacturer, differences between the active ingredients described could be identified. In addition, by selecting different manufacturers who all marketed the same active ingredients, differences between the individual manufacturers became apparent. To generate as large a database as possible, no specific exclusion criteria for the selection of certain PIs were defined. Online available PIs with both identical and different active ingredient concentrations were included to determine a correlation between the mentioned ADRs and the drug dosage.

The information from the package inserts were analysed according to the following criteria: named oral adverse drug reaction, associated prevalence with which the ADR occurs. The ADRs and their stated frequencies were then compared with each other, and differences between the PIs were analysed. The coded manufacturers and the medicinal products issued by the manufacturers, including PIs, are shown in Table 2.

**Table 2:** Presentation of the numerically coded pharmaceutical manufacturers with the respective analysed package inserts of the selected active ingredients

Manufacturer	Examined drug
1	Carbamazepine, valproic acid
2	Carbamazepine, amlodipine, felodipine
3	Carbamazepine, amlodipine, ciclosporin
4	Carbamazepine, phenytoin, valproic acid
5	Carbamazepine, amlodipine, felodipine
6	Carbamazepine, valproic acid, amlodipine
7	Carbamazepine, amlodipine, felodipine
8	Carbamazepine, amlodipine, ciclosporin
9	Phenytoin, ciclosporin
10	-
11	Valproic acid, ciclosporin
12	Valproic acid, felodipine, ciclosporin
13	Valproic acid, felodipine
14	-
15	Valproic acid, amlodipine, felodipine, ciclosporin
16	Valproic acid, amlodipine, felodipine
17	Valproic acid, amlodipine, felodipine, ciclosporin
18	Amlodipine, felodipine
19	Amlodipine
20	Amlodipine
21	Amlodipine

The drugs analysed can basically be divided into the following drug classes: anticonvulsants, calcium channel blockers, immunosuppressants and others. In addition, exemplar comparisons were made between package insert data and SmPC information. The aim of this comparison was to find out whether the SmPCs provide more precise information on DIGO or interactions between different active substances, treatment options or a possible regression of the adverse reaction when the drug is discontinued. Subsequently, the current prescription figures from the AVR 2023 of some drugs were compiled in order to determine a model calculation for the evaluation of the patients with DIGO to be expected per year with the help of the prevalences given in the package inserts.

## Results and discussion

### Anticonvulsants

Among the 14 examined anticonvulsants, 11 showed no differences regarding the stated adverse drug reactions inside the package inserts. In contrast, the PIs of carbamazepine, phenytoin and valproic acid contained the following variations (Table 3).

**Table 3:** Overview of the number of package inserts analysed for each active substance among the group of anticonvulsants, as well as the adverse drug reactions mentioned with their associated prevalence. The investigated substances are carbamazepine, phenytoin, valproic acid, gabapentin, eslicarbazepine, phenobarbital, primidone, topiramate, ethosuximide, lamotrigine, vigabatrin and mesuximide

Number of package inserts	Stated adverse drug reaction	Prevalence
Carbamazepine		
18	Gingivitis	Very rare
7	Gingivitis	Individual cases
2	Inflammation of the mucous membranes of the mouth and oropharynx	Very rare
1	Inflammation of the oral mucosa, gums and tongue mucosa	Individual cases
2	Inflammation of the oral mucosa, gums and tongue mucosa	Very rare
Phenytoin		
2	Gingival hyperplasia	Uncommon
1	Gingival overgrowth	Uncommon
Valproic acid		
4	Gum disease (mainly gingival overgrowth)	Common
2	Stomatitis	Rare
2	No gum-related adverse drug reactions	/
16	Gum disease (mainly gingival overgrowth), inflammation of the oral mucosa	Common
Gabapentin		
20	Gingivitis	Common
Eslicarbazepine		
22	Gingivitis or toothache	Uncommon
Phenobarbital		
5	Gingival hyperplasia	Not known
Primidone		
4	No gum-related adverse drug reactions	/
Topiramate		
15	Bleeding gums	Uncommon
Ethosuximide		
7	No gum-related adverse drug reactions	/
Lamotrigine		
20	No gum-related adverse drug reactions	/
Vigabatrin		
14	No gum-related adverse drug reactions	/
Mesuximide		
2	No gum-related adverse drug reactions	/

For a better understanding, the results of Table 3 are described below exemplarily. The same principle applies to Tables 4, 5 and 6, but for reasons of brevity, a repeated description of the results of these tables has been skipped.

In total, 30 package inserts of carbamazepine were examined. Eighteen stated “gingivitis” as a “very rare” adverse drug reaction; seven stated “gingivitis” as an adverse drug reaction in “individual cases”; two inserts stated “Inflammation of the mucous membranes of the mouth and oropharynx” as a “very rare” adverse reaction; one stated “inflammation of the oral mucosa, gums and tongue mucosa” in “individual cases”. Two other inserts stated “inflammation of the oral mucosa, gums and tongue mucosa” as a “very rare” adverse reaction.

In total, three package inserts of phenytoin were examined. Two stated “gingival hyperplasia” as an “uncommon” adverse drug reaction; one stated “gingival overgrowth” as an “uncommon” adverse drug reaction.

In total, 24 package inserts of valproic acid were examined. Four inserts stated “gum disease (mainly gingival overgrowth)” as a “common” adverse drug reaction; two inserts stated “stomatitis” as a rare adverse drug reaction; in two other inserts, no gum-related adverse drug reactions were mentioned. Sixteen inserts stated “gum disease (mainly gingival overgrowth), inflammation of the oral mucosa” as a common adverse drug reaction.

In addition, current literature indicates that DIGO is exacerbated by the simultaneous use of various anticonvulsants and a combination of immunosuppressants (e.g. ciclosporin) and calcium channel blockers (e.g. nifedipine) (Tungare and Paranjpe 2022; Thomason et al. 1997; Khoori et al. 2003). A large number of medications are suspected of influencing the severity of gingival overgrowth when taken at the same time. These include, for example, the simultaneous use of ciclosporin and azathioprine, as well as ciclosporin and calcium channel blockers such as amlodipine (Dongari-Bagtzoglou 2004). It has been shown that prednisolone and azathioprine have a positive effect on gingival tissue (Wilson et al. 1998), while the simultaneous use of ciclosporin and amlodipine significantly increases the likelihood of developing gingival overgrowth (James et al. 2000). In the PIs analysed for all the drugs described in this paper, only the information of ciclosporin contained a reference to a mutual drug interaction. There is a warning that taking nifedipine in addition to ciclosporin could lead to swelling of the gums with possible overgrowth of the teeth. The package inserts for nifedipine did not contain any such information regarding the simultaneous use of ciclosporin and nifedipine. In principle, the PIs only contained the information that the simultaneous intake of different drugs can lead to a mutual interaction, which can also lead to an aggravation of the possible ADRs of the individual active substances. The active substance groups in focus (anticonvulsants, calcium channel blockers, immunosuppressants) are listed in the investigated package inserts, and a reciprocal interaction when taken at the same time is also described, but there is no specific reference to a possible worsening of drug-induced gingival overgrowth. In addition, the PIs of various calcium channel blockers contained the information that the observed gingival overgrowth would decrease after discontinuation of the therapy. This information was not found in any of the package inserts of other drug classes. There is a further detail in the felodipine package insert, which explicitly recommends consulting a doctor or pharmacist if there is already swelling of the gums, e.g. due to periodontitis. In addition, according to the package insert, careful dental hygiene may be necessary to avoid further gum problems. Nevertheless, there is no evidence of therapeutic procedures to combat gingival overgrowth in the PIs analysed.

### Calcium channel blockers

Among the 13 examined calcium channel blockers, 6 showed no differences regarding the stated adverse drug reactions. Amlodipine, felodipine, nitrendipine, verapamil, nicardipine, nisoldipine and lercanidipine, respectively, showed the following variations (Table 4).

**Table 4:** Overview of the analysed package inserts of calcium channel blockers, the adverse drug reactions and prevalences mentioned therein. The investigated substances are amlodipine, felodipine, nifedipine, nitrendipine, diltiazem, verapamil, nicardipine, nisoldipine, isradipine and lercanidipine

Number of package inserts	Stated adverse drug reaction	Prevalence
Amlodipine		
2	Gingival hyperplasia	Very rare
15	Swelling of the gum	Very rare
9	Bleeding and swelling of the gum	Very rare
Felodipine		
6	Bleeding and slight swelling of the gums	Very rare
1	Gingivitis, swollen gums	Very rare
7	Gingival overgrowth and gingivitis	Very rare
Nifedipine		
12	Gingival hyperplasia	Rare
Nitrendipine		
5	Gingival hyperplasia	Very rare
1	Gingival hyperplasia	Individual cases
5	Gingival hyperplasia	Uncommon
1	No gum-related adverse drug reactions	/
Diltiazem		
9	Gingival hyperplasia	Very rare
Verapamil		
12	Gingival hyperplasia	Not known
5	Gingival hyperplasia, gingivitis, bleeding gums	Very rare
Nicardipine		
1	Gingival hyperplasia	Not known
1	Gingival hyperplasia	Individual cases
1	No gum-related adverse drug reactions	/
Nisoldipine		
1	Gingival hyperplasia	Rare
1	Gingival hyperplasia	< 1%
1	Gum changes	Rare
Isradipine		
2	Gingival hyperplasia	Rare
Lercanidipine		
1	No gum-related adverse drug reactions	/
1	Swollen gums	Not known
1	Gingival hyperplasia	Very rare

### Immunosuppressants

Among the five examined immunosuppressants, three showed no differences regarding the stated adverse drug reactions. In contrast, ciclosporin and methotrexate showed the following variations (Table 5).

**Table 5:** Overview of the analysed package inserts of immunosuppressants, the adverse drug reactions and prevalences mentioned therein. The active ingredients considered here are ciclosporin, tacrolimus, sirolimus, methotrexate and cyclophosphamide

Number of package inserts	Stated adverse drug reaction	Prevalence
Ciclosporin		
15	Could lead to gingival hyperplasia if nifedipine is taken simultaneously	Common
1	Gingivitis and gingival overgrowth, nifedipine should not be taken simultaneously	Common
Tacrolimus		
12	No gum-related adverse drug reactions	/
Sirolimus		
2	Stomatitis	Common
Methotrexate		
1	Gingivitis	Rare
2	Stomatitis, oral ulcerations, gingivitis	Very common, common, rare
Cyclophosphamide		
1	Stomatitis	Common

### Other drugs

Among the four examined drugs belonging to different groups of medication, three showed no differences regarding the stated adverse drug reactions (Table 6). For diphenoxylate, the following variations were found.

**Table 6:** Overview of the analysed package inserts, the adverse drug reactions and prevalences mentioned therein. The investigated substances belong to different groups of medication and are tranexamic acid, diphenoxylate, atropine and erythromycin

Number of package inserts	Stated adverse drug reaction	Prevalence
Tranexamic acid		
2	No gum-related adverse drug reactions	/
Diphenoxylate		
1	No gum-related adverse drug reactions	/
1	Red or swollen gums	Common
Atropine		
2	No gum-related adverse drug reactions	/
Erythromycin		
3	No gum-related adverse drug reactions	/

**Fig. 2:**
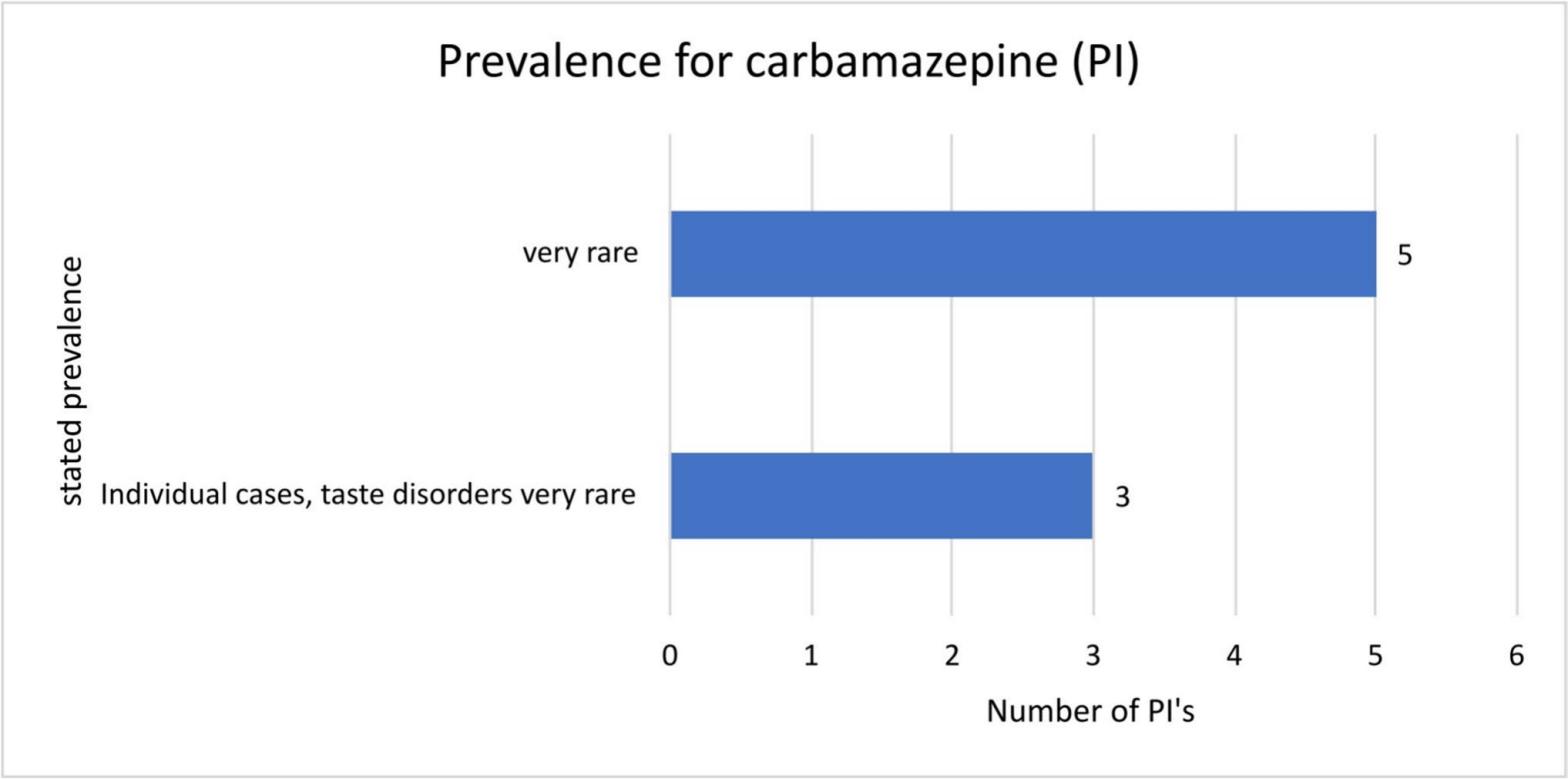
Overview of the prevalence of “stomatitis, gingivitis, glossitis, taste disorders” mentioned in the package inserts for carbamazepine and the number of corresponding package inserts

Regarding the PIs of the various manufacturers of carbamazepine, they all mention the same ADRs. However, there are differences in the stated frequencies (Fig. 2). Manufacturers 1–5 state that the ADRs mentioned occur “very rarely”, while manufacturers 6–8 describe that “stomatitis”, “gingivitis” and “glossitis” can occur in “individual cases”, but “taste disorders” are “very rare”. To summarise for carbamazepine, it can be stated that there are differences within the package inserts of different manufacturers. The reference to “additional” adverse drug reactions in individual sources and the different prevalences lead to uncertainty and loss of confidence on the part of both patients and treating physicians. As only a selection of manufacturers was analysed here, it is important to consider how large or frequent the differences could be if all manufacturers of an active ingredient are considered.

The package inserts of manufacturers 4 and 9 of phenytoin also showed no differences in terms of adverse drug reactions and frequencies (Table 7). It is striking that the package inserts for phenytoin refer to a connection between poor oral hygiene and the development of gingival overgrowths. This practically important reference is missing in almost all other PIs analysed.

**Table 7:** Presentation of the manufacturers of phenytoin with the adverse drug reactions mentioned in the PIs and the associated prevalence

Drug	Manufacturer	ADR PI	Prevalence PI
Phenytoin	4	Gingival overgrowth	Occasionally, gingival overgrowth occurs more frequently in children, adolescents and patients with poor oral hygiene
	9	Gingival overgrowth	Occasionally, gingival overgrowth occurs more frequently in children, adolescents and patients with poor oral hygiene

**Fig. 3:**
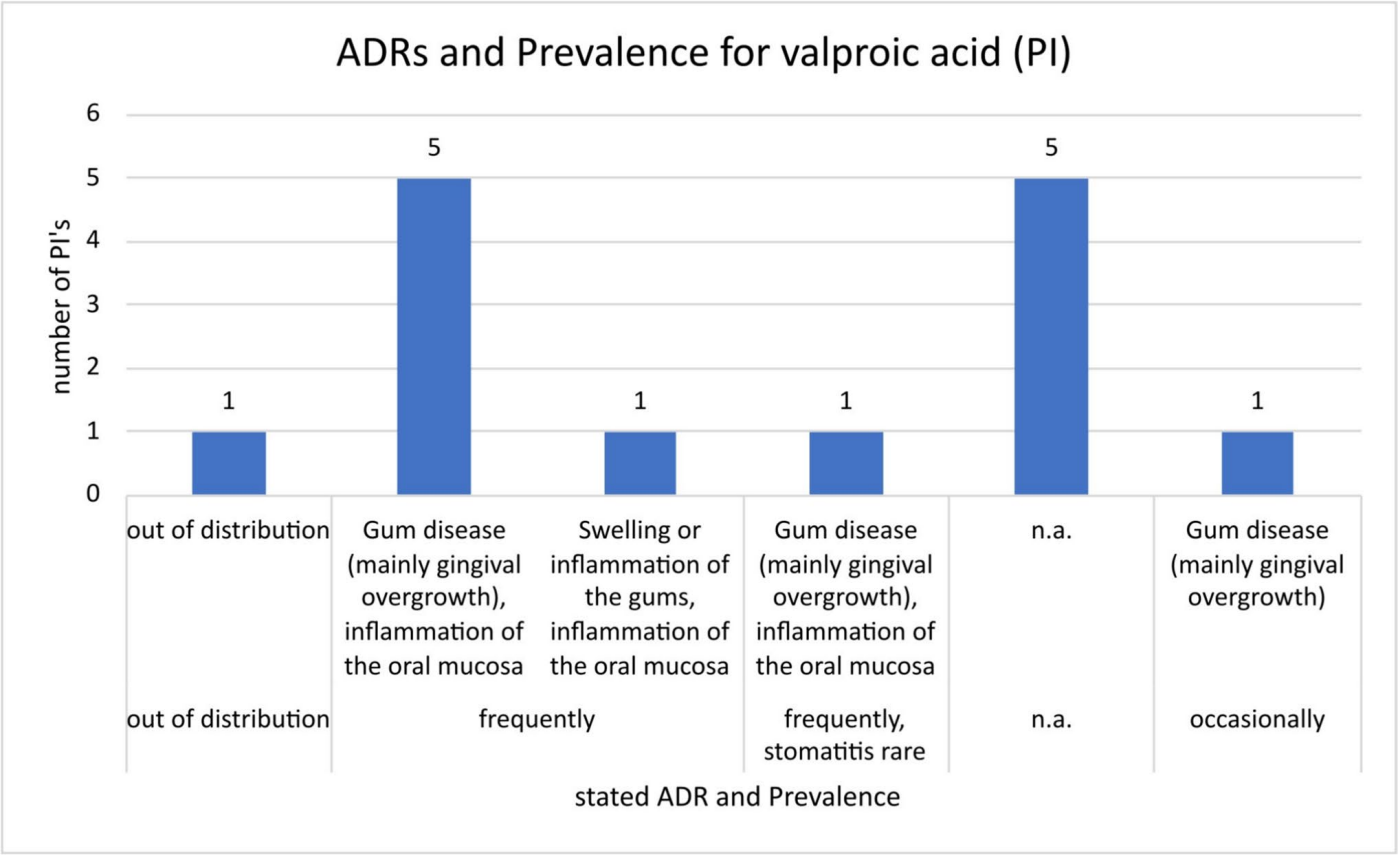
Presentation of the number of different package inserts of valproic acid with the adverse drug reactions mentioned therein and the associated prevalence

The package inserts for valproic acid from manufacturers 10, 5, 14, 2 and 18 were not available at the time of the study. The preparation from manufacturer 11 was out of distribution at the time of the study. In the PI of manufacturer 12, “gum disease (mainly gingival overgrowth)” was mentioned as an ADR with “occasional” frequency (Fig. 3). The PIs of manufacturers 4, 13, 16, 6, 17 and 1 differ from the previous one in that the frequency stated is “frequently” and the adverse drug reaction described is “gum disease (mainly gingival overgrowth), inflammation of the oral mucosa”. The package insert from manufacturer 6 also refers to “stomatitis”, which is said to occur “rarely”. The ADRs mentioned by manufacturer 15 are “swelling or inflammation of the gums, inflammation of the oral mucosa”, also with the frequency “frequently”.

**Fig. 4:**
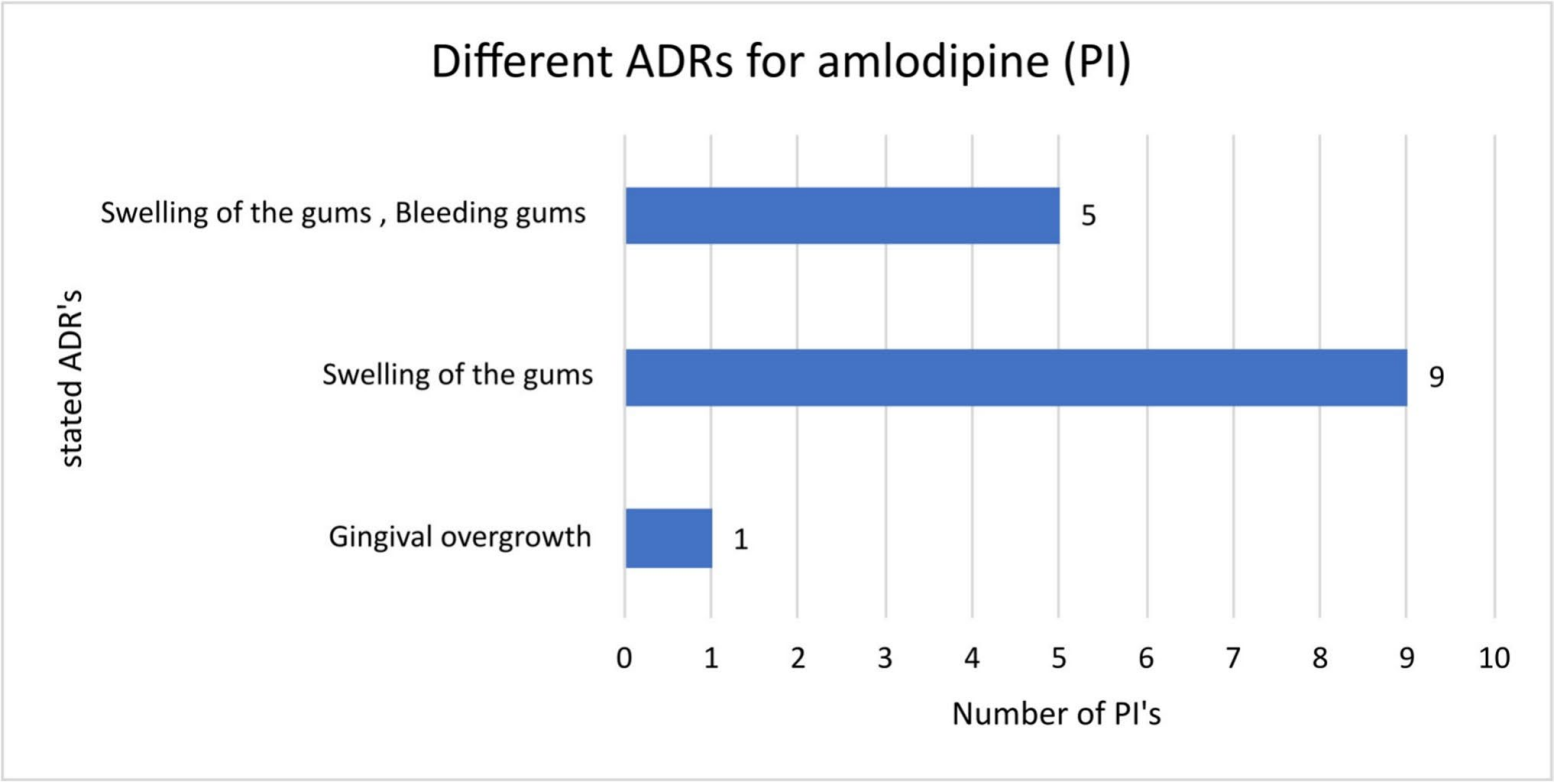
Number of different package inserts of amlodipine with the ADRs mentioned therein

The PIs for amlodipine from manufacturers 19, 20, 15, 5, 18, 17, 7, 12 and 3 differed neither in the adverse drug reactions mentioned nor in their frequency (Fig. 4). Manufacturers 16, 6, 2, 21 and 8 also mentioned “bleeding gums” as a possible ADR in comparison to the previously analysed manufacturers. The PI from manufacturer 8 was the only one stating “gingival overgrowth” as an ADR. The frequencies mentioned here were also identical.

**Fig. 5:**
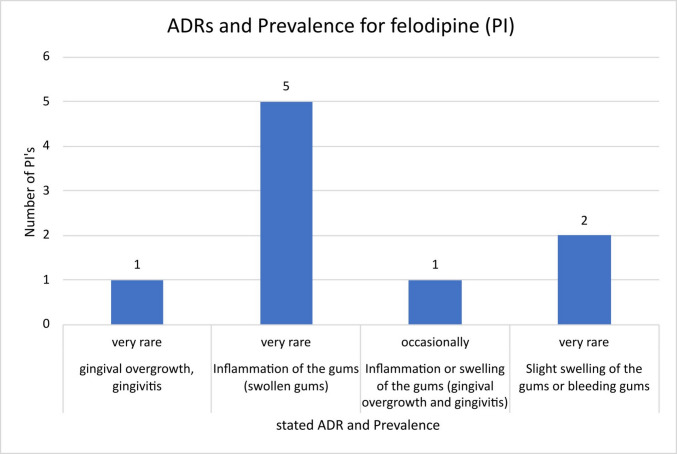
Overview of the number of different PIs of felodipine with the adverse drug reactions mentioned therein and the corresponding prevalence

For felodipine, except for manufacturer 18, who stated “occasionally”, all other manufacturers analysed stated “very rare” as the frequency in their package inserts (Fig. 5). Looking at the adverse drug reactions mentioned, the following differences stand out: manufacturers 12 and 13 mentioned “slight swelling of the gums or bleeding gums” as an ADR. Manufacturers 17, 15, 7, 16 and 2 mentioned “inflammation of the gums (swollen gums)”, while manufacturer 5 stated “gingival overgrowth, gingivitis” and manufacturer 18 “inflammation or swelling of the gums (gingival overgrowth and gingivitis)”. The differences described relate to the severity of the swelling and the presence of an inflammatory component.

**Fig. 6:**
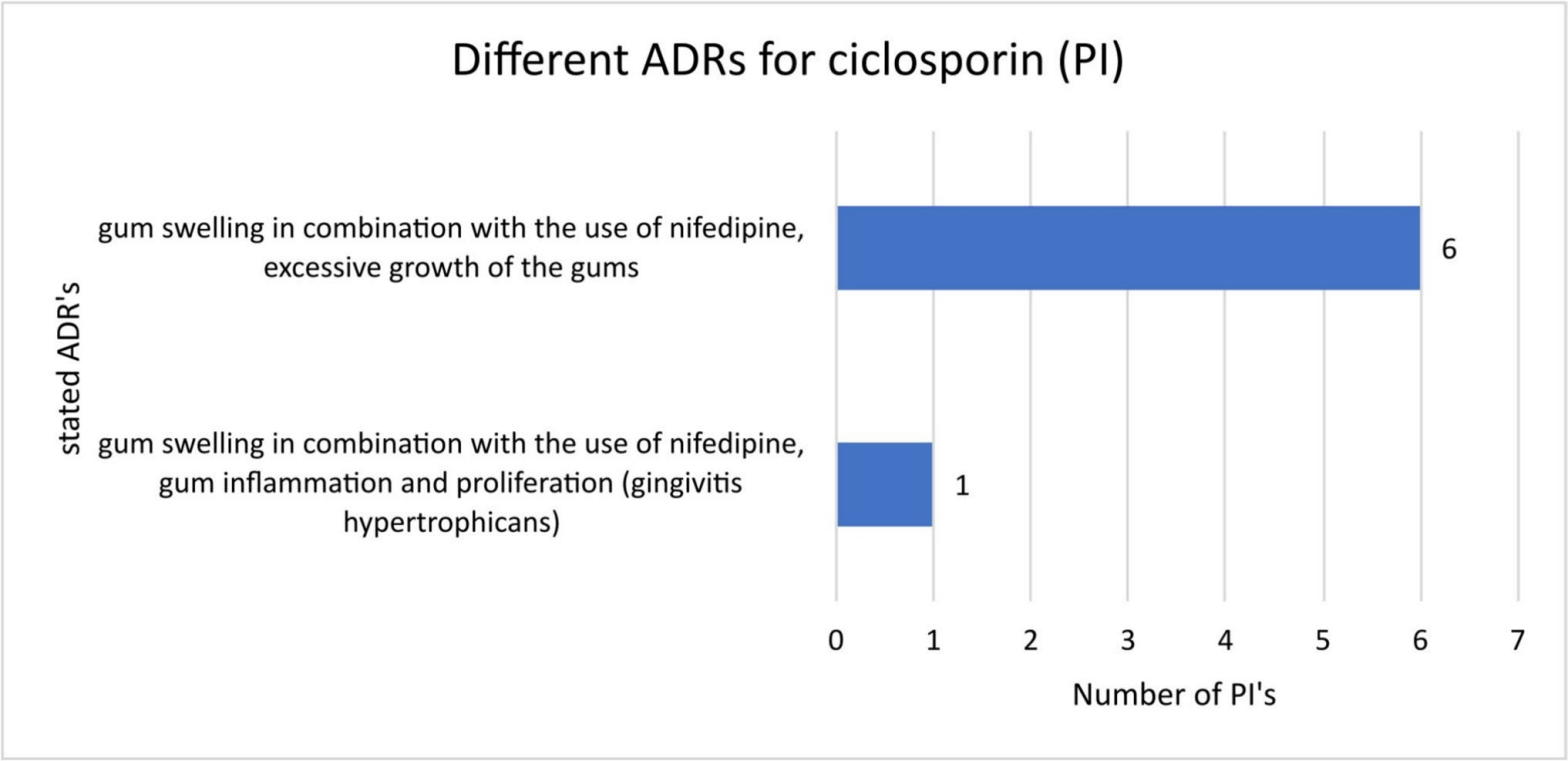
Presentation of the number of package inserts of ciclosporin with the ADRs mentioned therein

The analysed PIs for ciclosporin from manufacturers 17, 15, 8, 9, 12 and 3 showed no differences regarding the ADRs mentioned and their prevalences (Fig. 6). Only manufacturer 11 additionally referred to “gum inflammation” in the mentioned adverse drug reactions. In all the PIs, the wording of the ADRs sounds as if gum inflammation can only occur if nifedipine is taken simultaneously. Therefore, it could be concluded that taking ciclosporin alone would not lead to such proliferation.

In contrast to the assumption that more detailed information on the topic of drug-induced gingival overgrowth is provided in the information for healthcare professionals compared to the package inserts, this exemplary selection of drugs shows comparably imprecise content in both sources (Table 8). No differences in content could be found between the package inserts and SmPCs of carbamazepine, phenytoin, ciclosporin, amlodipine and nifedipine. A decrease in gingival overgrowth despite continuation of treatment was found in the SmPC of valproic acid, whereas no evidence of this was found in the corresponding PI. In the SmPC of felodipine, a prevention or regression of DIGO through particularly thorough dental hygiene is described, while no indication of this is found in the PI. In contrast, the PIs of nicardipine and nisoldipine state that gingival overgrowth regresses after discontinuation of the drugs, while this information is completely missing in the corresponding SmPCs. In summary, it is concerning that information for healthcare professionals are sometimes more imprecise and lacking relevant information in comparison to package inserts.

**Table 8:** Selection of active substances with the information on DIGO mentioned in the package inserts and SmPCs in comparison with each other

Drug	Stated information package insert	Stated information SmPC
Carbamazepine	Individual cases of abdominal pain and inflammation of the mucous membranes in the mouth and throat area (stomatitis, gingivitis, glossitis) have been reported	Individual cases of abdominal pain and inflammation of the mucous membranes in the mouth and throat (stomatitis, gingivitis, glossitis) have been reported
Phenytoin	Occasionally, gingival hyperplasia (growth of the gums) occurs. The adverse reaction profile of phenytoin is generally similar in children and adults. Gum growths (gingival hyperplasia) occur more frequently in children, adolescents and patients with poor oral hygiene	The adverse reaction profile of phenytoin is generally similar in children and adults. Gingival hyperplasia (gum growths) occurs more frequently in paediatric patients and patients with poor oral hygiene. Occasionally gingival hyperplasia occurs
Valproic acid	Gum disease (mainly gum overgrowth), inflammation of the oral mucosa (sores, swelling, ulcers and burning sensation in the mouth)	Vomiting* 1, gum disease (mainly gingival hyperplasia), stomatitis, diarrhoea* 1, stomach pain* 1, which usually resolved after a few days despite continuing treatment. * 1 Especially at the beginning of treatment
Ciclosporin	Nifedipine is used to treat high blood pressure and heart pain. Gingival overgrowth could occur and the gums could overgrow your teeth if you use nifedipine during your treatment with ciclosporin. Excessive growth of the gums covering the teeth	Gingival hyperplasia. The use of nifedipine together with ciclosporin can lead to an increased rate of gingival hyperplasia compared to the use of ciclosporin alone
Tacrolimus	/	/
Amlodipine	Swelling of the gums	Gingival hyperplasia
Felodipine	Inflammation or swelling of the gums	As with other calcium antagonists, mild enlargement of the gingiva has been reported in patients with severe gingivitis/periodontitis. The enlargement can be prevented or reversed by careful dental hygiene. Gingival hyperplasia and gingivitis
Nifedipine	Gum changes (gingival hyperplasia) may occur during prolonged treatment with nifedipine, which disappear completely after discontinuation of therapy	Prolonged treatment with nifedipine can lead to changes in the gums (e.g. gingival hyperplasia), which disappear completely after discontinuation of therapy
Nicardipine	In individual cases, gum changes (gingival hyperplasia), enlargement of the male mammary glands (gynaecomastia) and hypersensitivity of the skin to light (photoallergic reactions) have been observed, which have regressed after discontinuation of the preparation	/
Nisoldipine	Gum changes (gingival hyperplasia; regresses after discontinuation of treatment)	Gingival hyperplasia
Azathioprin	/	/

### Regulations of PIs and SmPCs

When creating package inserts and SmPCs, the requirements of the European Union (EU) are the legal basis, implemented in national law. The respective guideline is “Directive 2001/83/EC”, which applies in Germany in the form of Sect. 11 of the German Medicines Act (§ 11 Arzneimittelgesetz) (AMG). Furthermore, pharmaceutical information in the package inserts and SmPCs must be consistent. The package leaflet should present the information in a way that is as understandable and legible as possible for every patient. In this context, the competent authorities in Germany are the BfArM and the Paul Ehrlich Institute (BfArM, 2025).

With regard to SmPCs, all ADRs observed in connection with the active substance should be listed in the relevant section. The most serious and most frequent adverse reactions should be listed first, followed by all others in descending order. Adverse reactions that were not explicitly observed in studies prior to the authorisation of the medicinal product but for which there is reasonable suspicion that the active substance could cause these ADRs should also be listed. Individual case reports, post-authorisation safety studies or spontaneous reports in which a causal relationship is at least a reasonable possibility are sufficient for this purpose.

With regard to frequencies, there are clear recommendations for calculating and categorising individual ADRs into the following groups: “very common (≥ 1/10)”; “common (≥ 1/100 to < 1/10)”; “uncommon (≥ 1/1,000 to < 1/100)”; “rare (≥ 1/10,000 to < 1/1,000)”; “very rare (< 1/10,000)” and, in exceptional cases, classification as “frequency not known” if no frequency can be calculated based on the available data. According to the EMA’s guidelines, the entire section of the SmPC dealing with ADRs should be “regularly reviewed” and updated if necessary. However, no specific time frame is stated for when regular reviews or revisions should take place. Nonetheless, the section may be revised at the latest when the marketing authorisation is renewed and as part of the Periodic Safety Update Reports (PSUR) that take place every 3 years (European Union 2025).

The marketing authorisation holder is also obligated to regularly review the safety of the active substance marketed with regard to package inserts. Even after marketing authorisation has been granted, appropriate studies should be conducted to verify safety. Moreover, the marketing authorisation holder is obligated to keep the information accompanying the medicinal product up to date with the latest scientific findings (European Union 2025).

The following model calculation is intended to illustrate how many patients per year would have to develop a DIGO under long-term therapy with various drugs if the prevalences stated in the package inserts are taken as a basis (Table 9). The prescribed defined daily dose (DDDs) for the relevant active substances was determined using the AVR for 2023 (Ludwig et al., 2023). Assuming that a patient in long-term therapy receives 365 DDDs per year, the number of total DDDs was divided by 365 to arrive at the number of patients. The number of patients treated with the respective drug in continuous therapy was multiplied by the prevalence stated in the package inserts to calculate the expected number of patients who would develop a DIGO under therapy. The following table shows the results sorted by active substance starting with the highest prescription quantity.

**Table 9:** Overview of various drugs with the associated DDDs, the number of patients receiving the corresponding drug in long-term therapy and the expected cases of DIGO calculated on the basis of the prevalences stated in the package inserts. The values shown refer to Germany. The prevalences given in the form of BfArM definitions from the package inserts were translated into decimal numbers, and the mean value was calculated for a given range (example, common corresponds to 1–10% mean value of 1–10% = 5.5%)

Drugs analysed	Recent DDDs in Germany	Number of patients with long-term therapy in Germany	Prevalence according to package insert	DIGO cases to be expected in Germany
Amlodipine	2,362,100,000	6,471,000	0.01%	647
Valproic acid	49,800,000	136,430	5.50%	7504
Verapamil	48,700,000	133,420	0.01%	13
Felodipine	41,400,000	113,420	0.01%	11
Carbamazepine	27,300,000	74,790	0.01%	7
Nifedipine	15,100,000	41,360	0.06%	25
Diltiazem	8,500,000	23,280	0.01%	2
Ciclosporin	6,600,000	18,080	5.50%	995
Phenytoin	3,800,000	10,410	0.55%	57

**Fig. 7:**
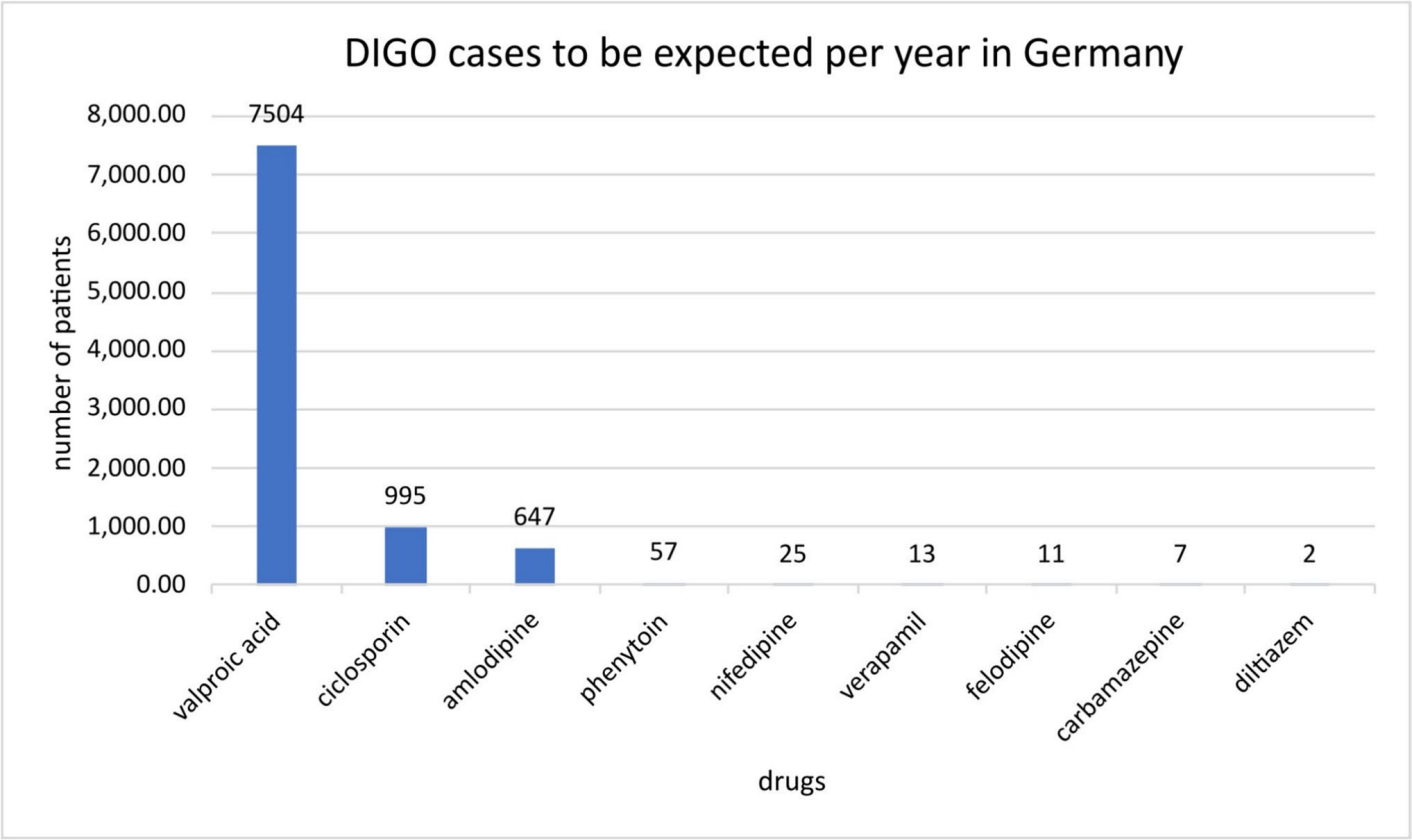
Illustration of the expected number of patients with DIGO per year in Germany depending on the respective drug taken in long-term therapy

The following illustration clearly shows that valproic acid in long-term therapy should lead to most cases of DIGO, assuming that the prevalence rates in the package inserts are correct (Fig. 7). The graph shows in descending order the number of patients who should develop DIGO per year under long-term therapy with the respective drug. Valproic acid, ciclosporin and amlodipine are by far the drugs that most frequently lead to DIGO. It is astonishing that the general perception is that phenytoin is much more closely associated with gingival overgrowth than valproic acid, even though valproic acid is prescribed much more frequently and has a tenfold higher prevalence of leading to gingival overgrowth. It should also be noted that although ciclosporin is prescribed significantly less frequently than amlodipine, it triggers DIGO more often than the calcium channel blocker.

It should be recognised that calculating the exact prevalence of ADRs is difficult. This can be attributed to various reasons. Different detection methods, reporting practices and population characteristics are described, leading to an extremely heterogeneous picture of the ADRs reported. This also explains the wide range between the presented prevalence rates (Pont et al. 2014). In addition, substantial under-reporting of ADRs is still being observed (Li et al. 2021). Moreover, patient-specific factors such as age, comorbidity and polypharmacy influence the frequency with which adverse drug reactions occur (Yadesa et al. 2021). Considering the different sizes of patient groups studied, it seems logical that ADRs occurring rarely or very rarely in particular are often not detected in correspondingly small study groups.

## Limitations

Only a limited number of active ingredients and a selection of manufacturers relevant for Germany were analysed in this study. Accordingly, there are many other manufacturers and active ingredients that were not taken into account here. Any changes to the package inserts between the time of data collection and publication of this article were not taken into account.

## Conclusions

To summarise, there are substantial differences within the PIs among the antiepileptic drugs, anticonvulsants and calcium channel blockers. The most serious of these are the partial lack of mentioning adverse drug reactions and significant differences in the stated prevalences. As all the data in the package inserts are obtained from studies conducted before the respective medication is authorised, the question arises as to how different results can arise. The studies may vary in precision, the underlying research question may vary or the analysis may be imprecise. Certain framework conditions must be established to ensure that both common and rare serious adverse drug reactions are detected and incorporated into the preparation of PIs in accordance with a standardised principle. The current legal framework allows too much flexibility to create a comparable basis. Although legislators describe various study designs that are suitable as a basis for the information in PIs and SmPCs, they do not provide specific guidelines for conducting these studies. For example, a specified minimum study size with a certain minimum number of participants could lead to rare adverse drug reactions being identified and their frequency estimated even before the active substance is approved for marketing. However, even after marketing authorisation, a re-evaluation of the expected ADRs with corresponding prevalences must be carried out at clearly defined intervals. This can lead to significant differences in the actuality of individual PIs and SmPCs, as currently the marketing authorisation holders are solely responsible for when and to what extent a review of the safety of the active substance is carried out. Accordingly, individual PIs and SmPCs may be significantly outdated compared to others. In addition, a uniform study design is recommended to produce reliable and comparable results.

Significant differences are also apparent in the context of post-market surveillance. For example, it is unclear to what extent data from various sources, such as spontaneous reporting databases, case studies or others such as social media, should be used to assess prevalences. This may lead to different weightings in the evaluation of these sources and correspondingly different calculated prevalences.

Finally, reformulating SmPCs to make them easier to understand for patients could result in imprecise and potentially incorrect statements in PIs. It is therefore mandatory that the readability check of the PIs also includes verification that no differences in content have arisen because of rephrasing or translation into another language.

Dissimilar information must be prevented in 100% of the cases to avoid uncertainty or, even worse, false information about certain drugs and their possible adverse reactions. Practitioners and patients need an even base of information to evaluate the risks of certain medications. Furthermore, it was shown that major differences between package inserts about the same active ingredient exist (Tables 3, 4, 5 and 6). PIs provide crucial information about the safety and instructions for use of the corresponding medication. It is unacceptable that different manufacturers provide dissimilar information about ADRs corresponding to the same active ingredient. Not only do the prevalence rates fluctuate between “common” and “unknown”, even the mentioned adverse reactions are unalike. Some package inserts do not even name some adverse reactions, whereas other inserts state a “common” prevalence. These results show that at the moment information presented in package inserts are not reliable and must be revised in the future.

Not only in Germany, but also in other parts of the world, such as the Middle East and Saudi Arabia, problems with the package inserts of various active ingredients can be identified (Gebran and al Haidari 2006). Differences were found between the information provided on one and the same drug in different countries, as well as between different manufacturers for the same active ingredient (Reggi et al. 2003). Therefore, it can be deduced that this is not a national problem, but various manufacturers from different countries are affected.

In addition to faulty PIs, SmPCs also lack important information on drug interactions between different active substances (Bergk et al. 2005). If this essential information is missing, patients may be harmed by the prescription of interacting active substances. Such gaps must be avoided, especially in information for healthcare professionals. However, it should be noted that the greatest differences between PIs were found with regard to the ADRs mentioned, as well as ambiguous and vague formulations (Storflor et al. 2013).

Another problem arises in the post-marketing surveillance of medicinal products. The fact that authorities and manufacturers are dependent on reports of spontaneously occurring ADRs in order to supplement them in package inserts and SmPCs, if necessary, results in an incomplete picture of adverse drug reactions (Tomita 2017). The question therefore arises as to how it can be ensured that all ADRs that occur can be registered as quickly as possible and included in all relevant information. In addition, regular monitoring and updating of the aforementioned information appear to be essential in order to incorporate the latest findings into patient medical care.

## Further perspectives

For future research, there is a growing list of drugs that are suspected of causing gum diseases, including gingival overgrowth, but there is no detailed review or confirmation of this link yet. In addition, the exact mechanisms of drug-induced gingival overgrowth have still not been conclusively clarified, so further studies are necessary to understand these complex relationships. Substantial differences between the PIs of different manufacturers should be identified and revised as quickly as possible to avoid confusion and possible non-adherence to the prescribed therapy (Bjerrum and Foged 2003). Package inserts are currently read by around 50% of patients on average. Factors that lead to an increase in this rate should be identified and implemented in newly published PIs. In addition, a negative effect on adherence caused by reading the PIs should be avoided, as well as frightening the patients (Vinker et al. 2007). The example of anticonvulsants can be used to illustrate that package inserts are often difficult to understand (Noble et al. 2022). The aim should therefore be to present the information relevant to patients in easily understandable language.

## Data Availability

All source data for this study are available upon reasonable request from the authors.

## Abbreviations

ADR: Adverse drug reaction
AVR: Drug prescription report (Arzneiverordnungs-Report 2023)
BfArM: Federal Institute for Drugs and Medical Devices (Bundesinstitut für Arzneimittel und Medizinprodukte)
CCB: Calcium channel blocker
DDD: Defined daily dose
DIGO: Drug-induced gingival overgrowth
GO: Gingival overgrowth
PI: Package inserts/package leaflets
SRP: Scaling and root planing
SmPC: Summary of product characteristics

## References

[CR1] Arning A, Seifert R (2023) Research letter. Dtsch Arztebl Int. 10.3238/arztebl.m2023.0153 37767580 10.3238/arztebl.m2023.0153 PMC10552627

[CR2] Arning A, Seifert R (2024) Insufficient correctness of package inserts for psychotropic drugs in Germany. Naunyn-Schmiedebergs Arch Pharmacol. 10.1007/s00210-024-03430-y 39302419 10.1007/s00210-024-03430-y PMC11919942

[CR3] Arya R, Gulati S, Kabra M, Sahu JK, Kalra V (2011) Folic acid supplementation prevents phenytoin-induced gingival overgrowth in children. Neurology 76(15):1338–1343. 10.1212/WNL.0b013e3182152844 21482950 10.1212/WNL.0b013e3182152844 PMC3090066

[CR4] Beaumont J, Chesterman J, Kellett M, Durey K (2017) Gingival overgrowth: Part 1: aetiology and clinical diagnosis. Br Dent J 222(2):85–91. 10.1038/sj.bdj.2017.71 28127024 10.1038/sj.bdj.2017.71

[CR5] Bergk V, Haefeli WE, Gasse C, Brenner H, Martin-Facklam M (2005) Information deficits in the summary of product characteristics preclude an optimal management of drug interactions: a comparison with evidence from the literature. Eur J Clin Pharmacol 61(5–6):327–335. 10.1007/s00228-005-0943-4 15983822 10.1007/s00228-005-0943-4

[CR6] Bharti V, Bansal C (2013) Drug-induced gingival overgrowth: the nemesis of gingiva unravelled. J Indian Soc Periodontol 17(2):182. 10.4103/0972-124X.113066 23869123 10.4103/0972-124X.113066 PMC3713748

[CR7] Bjerrum L, Foged A (2003) Patient information leaflets–helpful guidance or a source of confusion? Pharmacoepidemiol Drug Saf 12(1):55–59. 10.1002/pds.795 12616848 10.1002/pds.795

[CR8] Broich, K. (2015). Bekanntmachung von Empfehlungen zur Gestaltung von Packungsbeilagen nach § 11 des Arzneimittelgesetzes (AMG) für Humanarzneimittel (gemäß § 77 Absatz 1 AMG) und zu den Anforderungen von § 22 Absatz 7 Satz 2 AMG (Überprüfung der Verständlichkeit von Packungsbeilagen). Accessed: 2025, 27.02 (https://www.bfarm.de/SharedDocs/Bekanntmachungen/DE/Arzneimittel/natVerf/bm-zul-20150414-packungsbeilagen_2015-pdf.pdf?__blob=publicationFile)

[CR9] Brown, R., & Arany, P. (2015). Mechanism of drug‐induced gingival overgrowth revisited: a unifying hypothesis. Oral Diseases, 21(1). 10.1111/odi.12264 10.1111/odi.12264 PMC5241888 24893951

[CR10] Bundesinstitut für Arzneimittel und Medizinprodukte (BfArM) (n.d.). Packungsbeilage. Accessed: 2025, 30.09 (https://www.bfarm.de/DE/Aktuelles/Themendossiers/Packungsbeilage/_node.html)

[CR11] Chojnacka‐Purpurowicz J, Wygonowska E, Placek W, Owczarczyk‐Saczonek A (2022) Cyclosporine‐induced gingival overgrowth—review. Dermatol Ther. 10.1111/dth.15912 36208445 10.1111/dth.15912

[CR12] Dongari-Bagtzoglou A (2004) Informational paper: drug‐associated gingival enlargement. J Periodontol 75(10):1424–1431. 10.1902/jop.2004.75.10.1424 15562922 10.1902/jop.2004.75.10.1424

[CR13] Droździk A, Droździk M (2023) Drug-induced gingival overgrowth—molecular aspects of drug actions. Int J Mol Sci 24(6):5448. 10.3390/ijms24065448 36982523 10.3390/ijms24065448 PMC10052148

[CR14] European Union. (2025). RICHTLINIE 2001/83/EG DES EUROPÄISCHEN PARLAMENTS UND DES RATES. Accessed: 2025, 30.09 (https://eur-lex.europa.eu/legal-content/DE/TXT/HTML/?uri=CELEX:02001L0083-20250101#tit_1)

[CR15] Fuchs MD, Signer-Buset SL, Mendes S, Schmidt JC, Walter C (2019) Does systemically administered azithromycin have an effect on gingival overgrowth? A systematic review. Oral Surg Oral Med Oral Pathol Oral Radiol 128(6):606-614.e1. 10.1016/j.oooo.2019.07.022 31521584 10.1016/j.oooo.2019.07.022

[CR16] Gebran N, al Haidari K (2006) Assessment of prescribing information for generic drugs manufactured in the Middle East and marketed in Saudi Arabia. Ann Saudi Med 26(3):192–199. 10.5144/0256-4947.2006.192 16861873 10.5144/0256-4947.2006.192 PMC6074447

[CR17] James JA, Marley JJ, Jamal S, Campbell BA, Short CD, Johnson RWG, Hull PS, Spratt H, Irwin CR, Boomer S, Maxwell AP, Linden GJ (2000) The calcium channel blocker used with cyclosporin has an effect on gingival overgrowth. J Clin Periodontol 27(2):109–115. 10.1034/j.1600-051x.2000.027002109.x 10703656 10.1034/j.1600-051x.2000.027002109.x

[CR18] Khoori AH, Einollahi B, Ansari G, Moozeh MB (2003) The effect of cyclosporine with and without nifedipine on gingival overgrowth in renal transplant patients. Journal (Canadian Dental Association) 69(4):236–241 (**PMID: 12662462**) 12662462

[CR19] Li R, Curtis K, Zaidi STR, Van C, Thomson A, Castelino R (2021) Prevalence, characteristics, and reporting of adverse drug reactions in an Australian hospital: a retrospective review of hospital admissions due to adverse drug reactions. Expert Opin Drug Saf 20(10):1267–1274. 10.1080/14740338.2021.1938539 34077311 10.1080/14740338.2021.1938539

[CR20] Ludwig W-D, Mühlbauer B, Seifert R (2023) Arzneiverordnungs-Report 2023. Springer Berlin Heidelberg. 10.1007/978-3-662-68371-2

[CR21] Moffitt ML, Cohen RE (2013) Non-drug induced gingival enlargement. Gen Dent 61(5):e10–e13 (**PMID: 23928447**) 23928447

[CR22] Noble AJ, Haddad S, Coleman N, Marson AG (2022) Worth the paper they are printed on? Findings from an independent evaluation of the understandability of patient information leaflets for antiseizure medications. Epilepsia 63(8):2130–2143. 10.1111/epi.17299 35560228 10.1111/epi.17299 PMC9544238

[CR23] Pont, L., Alhawassi, T., Bajorek, B., & Krass, I. (2014). A systematic review of the prevalence and risk factors for adverse drug reactions in the elderly in the acute care setting. Clinical Interventions in Aging, 2079. 10.2147/CIA.S71178 10.2147/CIA.S71178 PMC4257024 25489239

[CR24] Reggi V, Balocco-Mattavelli R, Bonati M, Breton I, Figueras A, Jambert E, Kopp C, Montane E, Rägo L, Rocchi F (2003) Prescribing information in 26 countries: a comparative study. Eur J Clin Pharmacol 59(4):263–270. 10.1007/s00228-003-0607-1 12759794 10.1007/s00228-003-0607-1

[CR25] Storflor JG, Pettersen LC, Slørdal L, Spigset O (2013) Legemidlers pakningsvedlegg – ulik informasjon om like preparater. Tidsskr Nor Laegeforen 133(9):955–959. 10.4045/tidsskr.12.1272 23652143 10.4045/tidsskr.12.1272

[CR26] Thomason JM, Ellis JS, Kelly PJ, Seymour RA (1997) Nifedipine pharmacological variables as risk factors for gingival overgrowth in organ-transplant patients. Clin Oral Invest 1(1):35–39. 10.1007/s007840050006 10.1007/s007840050006 9552815

[CR27] Tomita T (2017) Research on insufficient information for pharmaceutical products. Yakugaku Zasshi 137(12):1497–1504. 10.1248/yakushi.17-00164 29199258 10.1248/yakushi.17-00164

[CR28] Tungare S, Paranjpe AG. Drug-induced gingival overgrowth. 2022 Sep 19. In: StatPearls. Treasure Island (FL): StatPearls Publishing; 2025. PMID: 30860753 Bookshelf ID: NBK538518 30860753

[CR29] Vinker S, Eliyahu V, Yaphe J (2007) The effect of drug information leaflets on patient behavior. The Israel Medical Association Journal : IMAJ 9(5):383–386 (**PMID: 17591379**) 17591379

[CR30] Wilson RF, Morel A, Smith D, Koffman CG, Ogg CS, Rigden SPA, Ashley FP (1998) Contribution of individual drugs to gingival overgrowth in adult and juvenile renal transplant patients treated with multiple therapy. J Clin Periodontol 25(6):457–464. 10.1111/j.1600-051X.1998.tb02474.x 9667479 10.1111/j.1600-051x.1998.tb02474.x

[CR31] Yadesa TM, Kitutu FE, Deyno S, Ogwang PE, Tamukong R, Alele PE (2021) Prevalence, characteristics and predicting risk factors of adverse drug reactions among hospitalized older adults: a systematic review and meta-analysis. SAGE Open Med. 10.1177/20503121211039099 34422271 10.1177/20503121211039099 PMC8377309

[CR32] Zoheir N, Hughes FJ (2019) The management of drug-influenced gingival enlargement. Prim Dent J 8(4):34–39. 10.1308/205016820828463816 10.1308/205016820828463816 32127092

